# Effect of Bulk Phase Composition on the Growth of PEO Coatings on the Biomedical Ti-6Al-4V Alloy

**DOI:** 10.3390/ma18050955

**Published:** 2025-02-21

**Authors:** José Roberto Ferreira Neto, Rafael Parra Ribeiro, Nilson Cristino da Cruz, Elidiane Cipriano Rangel, Bruna de Oliveira Pinto, Jhuliene Elen Muro Torrento, Carlos Roberto Grandini, Ulisses Ferreira Kaneko, Diego Rafael Nespeque Correa

**Affiliations:** 1 Laboratory of Technological Plasma, Institute of Science and Technology, Campus Sorocaba, São Paulo State University (UNESP), Sorocaba 18087-180, SP, Brazil; jose.roberto-ferreira@unesp.br (J.R.F.N.); rafael-parra.ribeiro@unesp.br (R.P.R.); nilson.cruz@unesp.br (N.C.d.C.); elidiane.rangel@unesp.br (E.C.R.); 2 Laboratory of Anelasticity and Biomaterials, School of Sciences, Campus Bauru, São Paulo State University (UNESP), Bauru 17033-360, SP, Brazil; bruna.oliveira-pinto@unesp.br (B.d.O.P.); jhuliene.torrento@unesp.br (J.E.M.T.); carlos.r.grandini@unesp.br (C.R.G.); 3 Institute of Geosciences and Exact Sciences, Campus Rio Claro, São Paulo State University (UNESP), Rio Claro 13506-900, SP, Brazil; ulisses.kaneko@unesp.br

**Keywords:** biomaterials, Ti-6Al-4V, heat treatment, phase composition, PEO, corrosion

## Abstract

This study investigated the effects of plasma electrolytic oxidation (PEO) treatment in a Ca- and P-rich electrolyte on the surface of the Ti-6Al-4V alloy with distinct α/β phase proportions previously induced by heat treatments. The results revealed that the α/β phase proportions were successfully altered by the heat treatment temperatures, forming α phase plates surrounded by β phase precipitates. PEO-treated samples exhibited a thick and microsized porous TiO_2_ coating in the anatase and rutile crystalline forms. The oxide layer was depleted by Al and V atoms, while Ca and P were gradually enriched along the coatings. Chemical analysis also indicated the absorption of water and organic molecules into the outer layer. PEO-treated samples had microscale roughness and thickness, hydrophilic behavior, and surface energy mainly formed by the dispersive component. The bulk’s elastic modulus decreased with β phase precipitation, while the alloying elements directly influenced the Vickers microhardness. The corrosion tests indicated a stable and protective layer in the PEO-treated samples, showing better corrosion resistance than untreated ones. Overall, the findings indicated that the α and β phase proportion significantly impacts the mechanical properties, while the PEO treatment acts in the corrosion protection and surface aspects, suggesting that combining both approaches could be a powerful tool in biomedical applications.

## 1. Introduction

Titanium (Ti) and its alloys have been extensively used as biomaterials due to their lower elastic modulus, recognized biocompatibility, and better corrosion resistance than conventional stainless steel and cobalt alloys [1]. Titanium’s biocompatibility is directly related to the chemical stability of the passive TiO_2_ layer naturally formed on its surface [2]. Moreover, the Ti-6Al-4V alloy (wt%, ASTM F136 [3], or commercially pure Ti grade 5) is currently the most popular Ti alloy worldwide, with versatile and tunable properties due to the coexistence of the α and β phases in the microstructure [4]. Nowadays, the material is still largely employed in manufacturing load-bearing orthopedic implants, despite the remaining concerns about the ion-releasing of harmful and toxic aluminum (Al) and vanadium (V) ions in long-term implantation [5]. Therefore, novel strategies for the surface modification of the Ti-6Al-4V alloy targeting attenuating corrosion in the human body are in demand.

Among the diverse methods for modifying metallic surfaces, plasma electrolytic oxidation (PEO), or micro-arc oxidation (MAO), is the most attractive for Ti and its alloys as it can produce a porous, thick, and firmly adherent TiO_2_ coating [6]. Based on the anodic oxidative reactions and the plasma discharges promoted by the dielectric breakdown, PEO treatment can significantly modify the surface topography, chemical composition, crystallinity, hydrophilicity, and roughness, which are key factors to consider in implant–tissue interaction and osseointegration [7]. Furthermore, the PEO treatment also permits the surface enrichment of bioactive elements, such as calcium (Ca) and phosphorous (P), through the driven force provided by the anodic voltage [8]. As is well known, calcium phosphate deposition on titanium surfaces has been a strategy used to make the implant’s surface friendlier to human bone tissues, enabling fast bone growth and recovery [9]. Even though there is vast amounts of research focused on the PEO treatment of Ti alloys [10,11], there is still a lack of a profound understanding of the role of the α and β phases on the coating’s growth mechanisms.

For the Ti-6Al-4V alloy, the alloying elements can decay the β-transus temperature (initially at 882 °C), making the control of the amount and distribution of α and β phases by proper heat treatments possible [12]. Previous studies have demonstrated their effectiveness for diverse industrial applications [13]. However, the role of the α and β phases on the oxide growth mechanisms during surface modification, especially PEO treatment, is scarcely reported. For example, Luz et al. [14] studied TiO_2_ nanotubes grown on the Ti-10Nb alloy using anodic oxidation treatment. The coating grown on the α phase depicted self-organized nanotubes, whereas that grown on the β phase presented some transversal holes. The α and β phases also affected wettability and P enrichment. Even though the applied voltage was not high enough to produce plasma discharges, the study provided new insights into developing biofunctional surfaces. In contrast, Yavari et al. [15] studied the effect of PEO treatment on the commercially pure Ti grade 2 (stable α), Ti-6Al-7Nb (α + β), Ti-35Zr-10Nb (metastable β), and Ti-45Nb (stable β) alloys in an electrolyte composed of calcium glycerophosphate (C_3_H_7_CaO_6_P) and calcium acetate (C_4_H_6_CaO_4_). The authors found that the pore characteristics (size and density) were larger for the β-type alloys, with the anatase TiO_2_ phase preferentially forming in the stable α- and β-type alloys in detriment of the metastable ones. However, the findings involving the growth mechanisms depended not exclusively on the phase composition but also on the alloying elements in solid solution.

Given that, the present study aimed to evaluate the effect of the α/β proportion of the Ti-6Al-4V alloy on the oxide layer growth by PEO treatment. The substrate underwent heat treatments at distinct temperatures and was then subjected to PEO treatment in a Ca- and P-rich electrolyte. After that, the chemical and phase composition, roughness, wettability, and mechanical and corrosion properties were evaluated for potential use in biomedical implants.

## 2. Materials and Methods

### 2.1. Sample Processing

**Figure 1:**
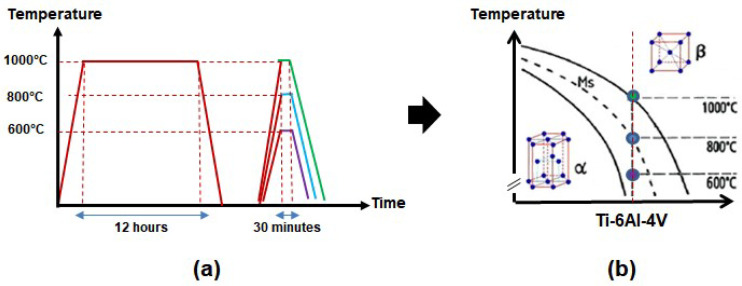
Processing route of the samples: (**a**) time–temperature plot and (**b**) the corresponding pseudo-binary phase diagram (adapted from [16]).

Square-shaped Ti-6Al-4V samples (20 × 20 × 3 mm) were used as substrates [3]. The samples were previously polished with waterproof SiC emery papers, from #220 to #1200 mesh, ultrasonically cleaned in acetone for 5 min, and dried in an air stream. The samples were subjected to an initial heat treatment for microstructural homogenization at 1000 °C for 12 h, with subsequent slow cooling in a turned-off furnace. Then, the samples were separated into three groups, which were heat-treated at 600 °C, 800 °C, and 1000 °C for 30 min, followed by fast water cooling (Figure 1a). All the heat treatments were performed at a vacuum of 10^−6^ Torr. The temperatures were set up to produce distinct amounts of α and β phases in the microstructure, as indicated in Figure 1b. The corresponding sample nomenclature is indicated in Table 1.

### 2.2. Surface Modification

The PEO treatment was conducted in the same experimental apparatus reported by Marcuz et al. [17]. In short, the surface treatment was carried out in a two-liter stainless steel tank, surrounded by a refrigeration system that maintained the electrolyte under constant temperature. The sample was connected to the positive terminal (anode) using a clamp, and the other terminal (cathode) was connected to the metal container. Then, the PEO treatments were carried out using a pulsed power source (MAO-30, Plasma Technology Ltd., Hong Kong, China) operated with positive pulses of 480 V, frequency of 100 Hz, and duty cycle of 60% for 120 s. The electrolyte was composed of 0.2 mol∙L^−1^ of calcium acetate (C_4_H_6_CaO_4_) and 0.02 mol∙L^−1^ of sodium glycerophosphate (C_3_H_7_CaO_6_P) in deionized water, being previously mixed by a magnetic stirrer. After that, the samples were cleaned with distilled water and dried in an air stream.

### 2.3. Sample Characterization

Microstructural and topographical characteristics were examined by optical microscopy (OM; BX51M microscope, Olympus Ltd., Westborough, MA, USA) and scanning electron microscopy (SEM; JSM-6010LA, JEOL Ltd., Peabody, MA, USA), using the secondary electron beam mode (SE), with a spot size of 30 µm, at a voltage of 3 kV. For the microstructural analysis, the selected part of the samples underwent conventional metallographic preparation based on grinding in waterproof SiC papers (#360 to #1500 mesh), polishing in alumina suspension (0.25 μm), and etching in Kroll’s solution. The phase composition was evaluated by X-ray diffraction measurement (XRD; Panalytical X’Pert PRO, Westborough, MA, USA), with monochromatic Cu-Kα radiation (λ = 0.1544 nm), at a voltage of 45 kV, a current of 40 mA, in the θ-2θ mode, with a scan speed of 0.02° per second, and a step size of 0.02°. The diffracted peaks were indexed by the Highscore Plus^®^ software version 3.0 and crystallographic datasheets from the ICDD (International Centre for Diffraction Data).

Semi-quantitative chemical microanalysis and elemental mapping were performed using X-ray dispersive energy spectroscopy (EDS) in a detector (JSM-6010LA, JEOL Ltd., USA) coupled to the SEM equipment operating at 10 kV. In the transmittance mode, the vibrational characteristics of molecular groups absorbed in the surfaces were analyzed using Fourier-transform infrared spectroscopy (FTIR; Jasco Corp., Tokyo, Japan) at room temperature, with 120 scans and a resolution of 4 cm^−1^. The vibrational characteristics of molecular absorbed groups in the surfaces were analyzed by Raman spectroscopy through an optical spectrometer (HRS300 model, Princeton Instruments Inc., Trenton, NJ, USA) set to a 1200 grooves/mm grating and equipped with a contact Peltier module (PIXIS100BR, Teledyne Princeton Instruments Inc., Trenton, NJ, USA). All spectra were collected in a confocal configuration with a 10× objective and a numerical aperture of 0.28 (Mitutoyo Inc., Aurora, IL, USA). The excitation source (Verdi 6V, Coherent Laser Corp., Saxonburg, PA, USA) was adjusted to 532 nm, with a power of 50 mW, and an accumulation time of 50 s. The surface chemical aspects were further investigated by X-ray photoelectron spectroscopy (XPS; K-Alpha model from Thermo Scientific Inc., Waltham, MA, USA), operating with monochromatic radiation source of Al-Kα (1486 eV), spot size of 400 μm, energy step of 200 eV for long-scan (survey) and 50 eV for short-scan spectra (high-resolution), with a resolution of 1 eV for long- and 0.01 eV for short-scan spectra. The CasaXPS^®^ software version 2.3.24 was used to evaluate the XPS spectra, with a previous energy calibration with the C1s peak.

Arithmetic roughness (Ra) was measured by optical profilometry (Dektak 150 profilometer, Veeco Metrology, Tucson, AZ, USA), with a stylus radius of 12.5 μm, scan length of 2000 μm, resolution of 0.16 μm, during 20 s, and applied load of 3 mgf. The surface wettability was checked by contact angle measurements (Ramé-hart Instrument Corp., Succasunna, NJ, USA) using droplets (30 µL) of distilled water, diiodomethane, and saline 0.9% NaCl solution at room temperature. The surface energy was calculated using Young’s equation [18]. Thickness values were estimated by the eddy current method (ME240 equipment, Instrutherm Inc., Sao Paulo, Brazil).

### 2.4. Sample Testing

The Vickers micro-hardness test was conducted in hardener equipment (Shimadzu Ltd., model HMV-G, Tokyo, Japan) operated at a load of 0.300 kg (2.942 N) and a dwell time of 15 s. The impulse excitation method acquired the elastic modulus using Sonelastic equipment (ATCP Physical Engineering Inc., Ribeirão Preto, SP, Brazil). The tests were repeated 10 times to collect reliable average values for each sample. The electrochemical tests were conducted in a three-electrode system, with the sample set up as a working electrode, a platinum rod (Pt) as a counter electrode, and an Ag/AgCl wire as a reference electrode. The experiments were carried out in an Autolab PGSAT128N potentiostat/galvanostat (Metrohm Ltd., Riverview, FL, USA). The electrolyte was composed of an aqueous 0.9% NaCl solution at room temperature, which is conventionally used to simulate the concentration of sodium chloride in human blood plasma [19]. The sample contact area with the electrolyte was limited by an O-ring (Ø = 1 cm). The open-circuit potential (OCP) was measured for 3600 s, while the potentiodynamic polarization (PDP) test was measured in the potential range of −1 to 2 V vs. OCP at a scan rate of 1 mV·s^−1^ and step size of 1 mV. Electrochemical impedance spectroscopy (EIS) measurements were performed by applying a perturbation of 10 mV concerning the OCP value in a frequency range from 10^−2^ to 10^5^ Hz, with 10 points per decade. The results were evaluated using NOVA^®^ software version 2.1.5, which also fitted the equivalent electrical circuit (EEC) of the curves. The corrosion potential (E_corr_) and corrosion current density (I_corr_) were calculated from the PDP curves using Tafel’s extrapolation method, with the polarization resistance (R_p_) obtained using the Butler–Volmer equation [20].

## 3. Results

**Figure 2:**
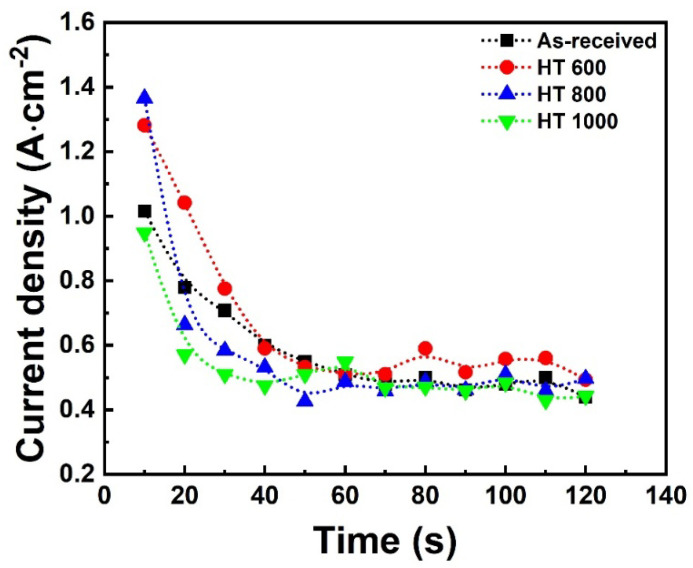
Current density vs. time plot for the Ti-6Al-4V samples in each condition.

Figure 2 shows the decay of the current density with the time for the samples during the PEO treatment. The current density sharply declined in the first seconds in all samples, starting at around 0.9–1.4 A·cm^−2^, followed by an exponential drop with some fluctuations after around 60 s, and ending between 0.4 and 0.5 A·cm^−2^. As is well known, the initial drop is related to the anodic growth of a dense oxide layer, which blocks the passage of electrical charges. The smooth decay and fluctuations are the result of establishing plasma discharges along the surface through the micro-arcs. Overall, the α and β phase proportions did not significantly affect the curves, suggesting that the alloying elements affect the growth kinetics more than the phase composition. Our previous study detected the same trend with the Ti-6Al-4V alloy when subjected to PEO treatment in a ZrO_2_-rich electrolyte and under the same electrical conditions [21].

**Figure 3:**
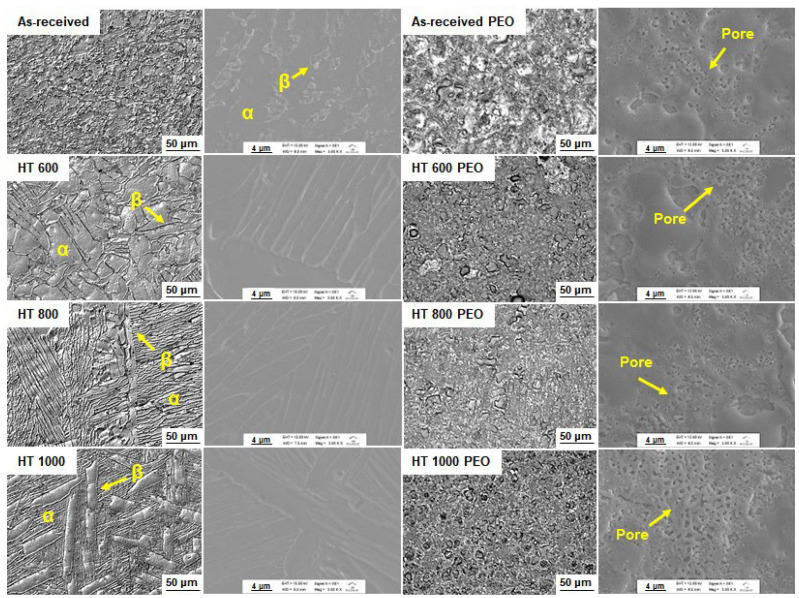
Microstructure and the corresponding surface topography of the samples: optical (**left**) and SEM (**right**) images.

Figure 3 shows the bulk microstructure and corresponding topography of the untreated and PEO-treated samples, respectively. The as-received sample possessed an irregular microstructure, with some β phase precipitates immersed in the α phase matrix. This microstructure is a result of previous plastic deformation performed by the supplier in the raw metal. The HT 600 sample depicted a partially recrystallized microstructure composed of typical α phase plates and some amount of β phase in the boundaries. As expected, the HT 800 sample presented a refined microstructure with thin α phase plates, indicating an augmenting of the β phase in the boundaries. Lastly, the HT 1000 sample possessed thinner α phase plates and coarse β phase precipitates, which the water cooling retained from the high temperature. Overall, the microstructures followed the expected α and β phase proportions by the distinct heat treatment temperatures, as illustrated in Figure 1. For the PEO-treated samples, a thick oxide layer with microsized porous structures without cracks or spallation can be seen, indicating that the α and β phases did not affect the global aspect of the topography, e.g., pore distribution and size. In our earlier study [22], Ti-15Zr-xMo (x = 0, 5, 10, and 15 wt%) alloys were subjected to a similar PEO treatment in a DC power source. The chemical and phase composition played a significant role in bioactivity, cell viability, mineralization, and differentiation ability, even though the topography remained almost the same.

**Figure 4:**
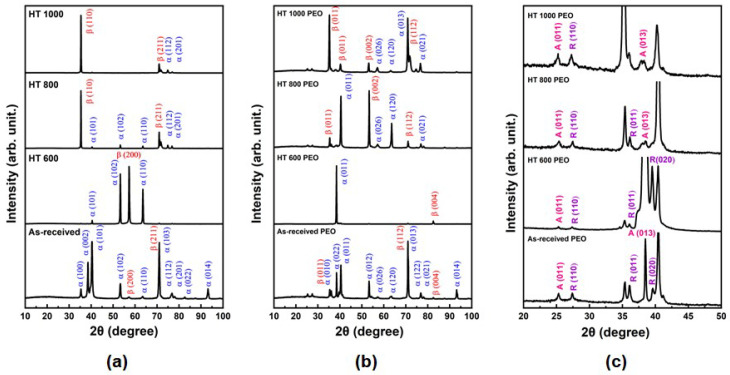
XRD profiles of the untreated (**a**) and PEO-treated (**b**) Ti-6Al-4V samples, with the corresponding zoomed-in view (**c**).

The XRD profiles in Figure 4 provide details about the phase composition of the bulk and surface. The untreated samples (Figure 4a) possessed a strong preferred orientation of some peaks from the α and β phases due to the previous plastic deformation of the raw samples. However, it is still possible to note that the as-received and HT 600 samples depicted major α and minor β phases, while the HT 800 and HT 1000 samples possessed a predominance of β phase. This result supports the microstructural modification previously shown in Figure 3. In the PEO-treated samples (Figure 4b), the peaks from the bulk phases were attenuated, indicating the growth of a thick oxide layer. In addition, it is possible to note some peaks from TiO_2_ as anatase (A) and rutile (R) phases. The A(101) and R(110) peaks remained at similar intensity in all sample conditions (Figure 4c), indicating that the bulk phase proportion did not present a major influence on the crystallinity of the coating. Similar results were found by Sobolev, Kossenko, and Borodianskiy [23] when studying the effect of current pulse frequency on the Ti-6Al-4V alloy subjected to PEO treatment in an AC power source and electrolyte enriched with Na_2_CO_3_ and Na_2_SiO_3_.

**Figure 5:**
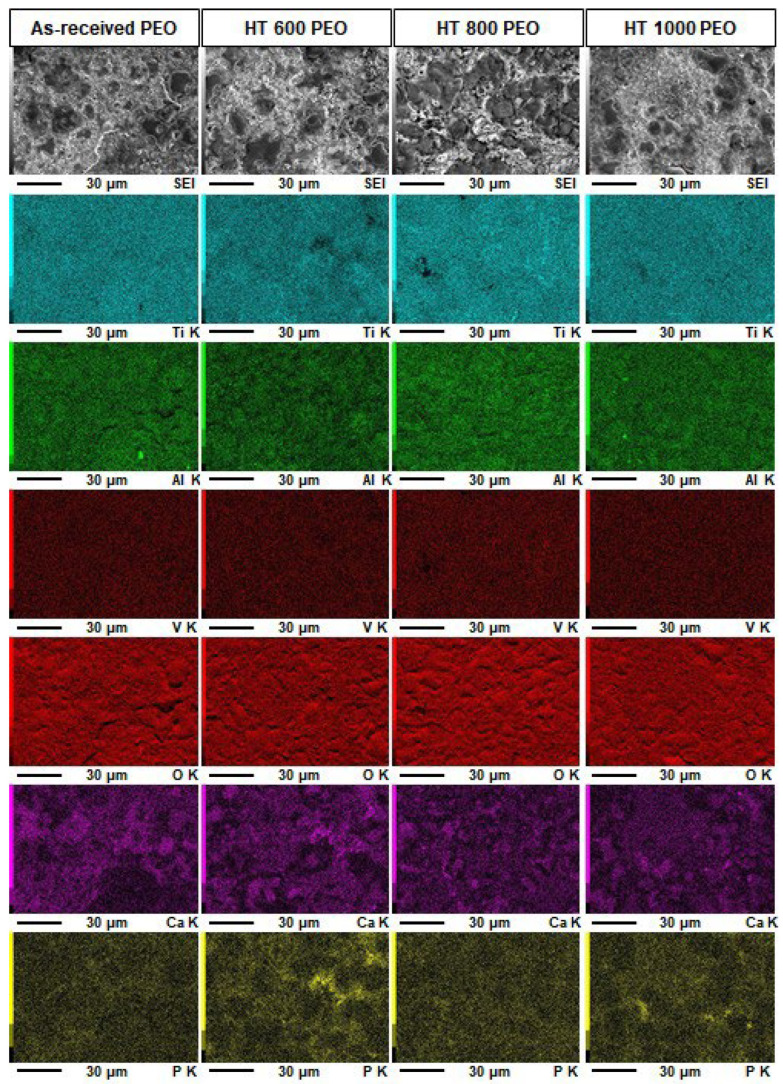
EDS analysis for PEO-treated Ti-6Al-4V samples.

Figure 5 shows the elemental mapping of the PEO-treated samples. The signal for Ti and O was more intense than that for Al and V, indicating a positive abundance of these elements in the coatings. The uniform distribution of Ti and O confirms a uniform coating after the PEO treatment. Ca and P atoms were detected in all samples without clear differences between the heat treatment conditions. Ca retained a more intense signal than P, suggesting a Ca/P ratio higher than 1.0, approaching that expected for hydroxyapatite (1.67). As already reported by previous studies, Ca- and P-rich PEO coatings on Ti surfaces can provide proper bioactivity through the calcium phosphate formation capability when immersed in body fluids [24].

**Figure 6:**
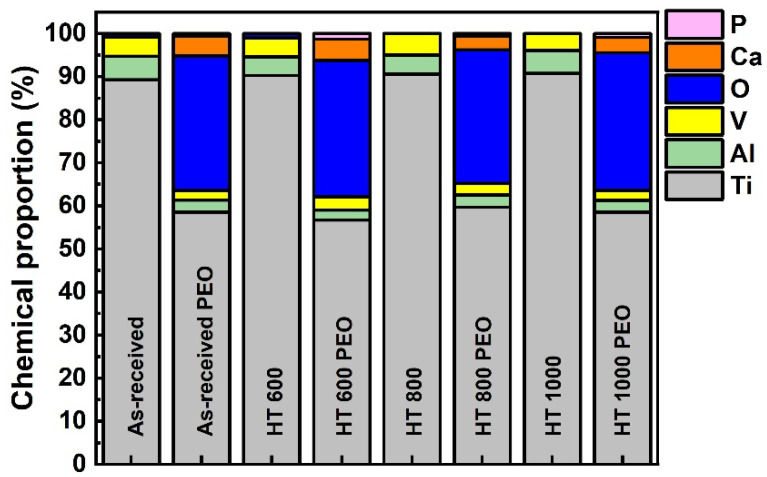
Semi-quantitative EDS analysis for Ti-6Al-4V samples in all studied conditions.

Figure 6 shows the corresponding semi-quantitative EDS analysis for the whole area of Figure 5 compared with the untreated samples. The PEO-treated samples possessed considerable oxygen due to the anodic oxide layer growth and a visible depletion of Al and V atoms compared to the bulk. This can positively avoid the release of harmful ions into the human blood. Ca atoms were detected in equal amounts and P remained as a trace, while the Ca/P ratio remained between 3.0 and 8.0. Overall, all heat-treated samples depicted the same chemical composition after PEO treatment when detected by EDS. However, considering the typical microsized thickness of the coatings and the considerable penetration depth of the electron beam, it can be presumed that the acquired chemical composition originates from bulk and surface signals [17].

**Figure 7:**
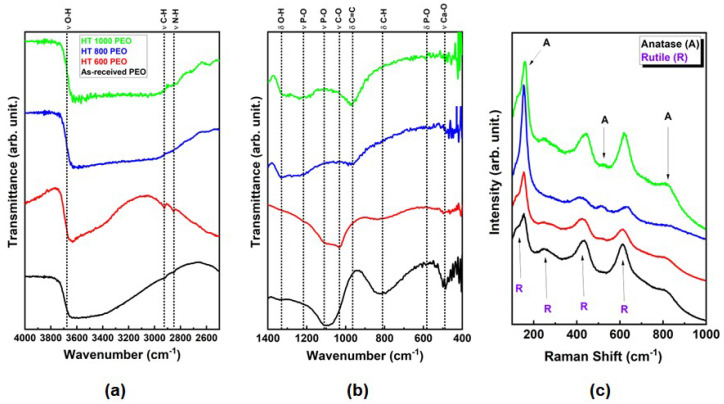
FTIR results at high (**a**) and low (**b**) wavenumbers and Raman spectra (**c**) for PEO-treated Ti-6Al-4V samples.

The FTIR and Raman spectra of the PEO-treated samples are presented in Figure 7. The FTIR spectra (Figure 7a,b) showed characteristic bands produced by stretching (ν) and bending (δ) of radical groups related to absorbed water (O-H), and organic (C-O, C=C, and C-H) and inorganic (N-H) molecules, and some related to phosphate (P-O) and calcium-based compounds (Ca-O). The Raman spectra depicted active vibration bands from TiO_2_ (anatase and rutile phases), with some distinct signal-to-noise ratio with the α and β phases. The bands in the Raman spectra were similar to those found in other PEO-treated Ti surfaces [23]. The presence of vibrational bands originating from Ca and P (FTIR) and TiO_2_ (Raman) shows that the PEO treatment successfully provided the growth of an anodic oxide layer rich in bioactive elements independently of the α and β phase proportions, which can guarantee favorable bioactive responses under implantation [25].

**Figure 8:**
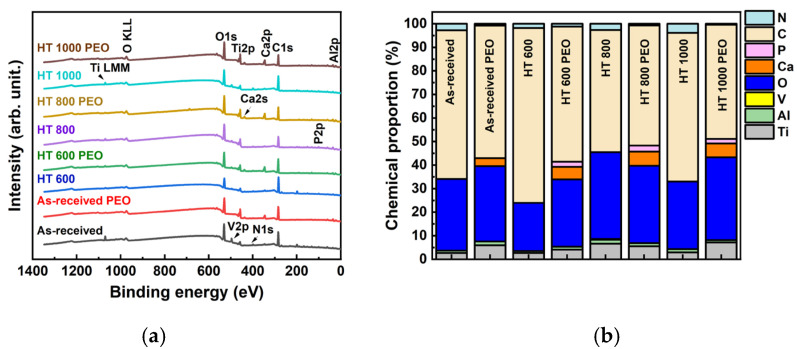
XPS semi-quantitative results: survey spectrum (**a**) and corresponding chemical composition (**b**) for Ti-6Al-4V in all studied conditions.

Figure 8a shows the survey spectra of all samples, where the main elements of interest are labeled. The semi-quantitative analysis (Figure 8b) presented a distinct chemical proportion compared to the EDS (Figure 6) due to the lower penetration depth of the X-ray beam. Thus, the outer layer comprised C from absorbed organic molecules and O from the oxide layer, as indicated in the FTIR (Figure 7a,b) and Raman spectra (Figure 7c), respectively. The PEO treatment also produced Ca enrichment of the outer layer, with a minor amount of P. The Ca/P ratio remained between 2.0 and 3.0, distinct from the EDS analysis, showing that the Ca and P atoms were not uniformly distributed into the coatings. The result also indicated a depletion of Al and a complete absence of V atoms in the outer layer, reinforcing the positive effect of the PEO treatment on the surface, as already highlighted in previous studies [26].

**Figure 9:**
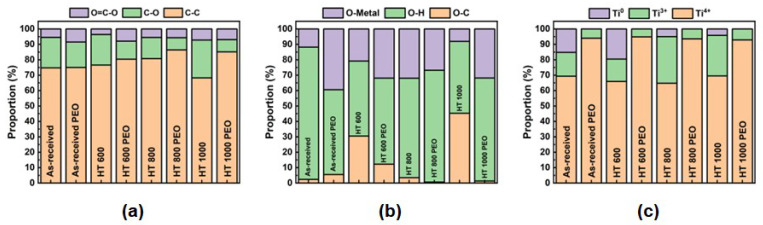
XPS HR results for C1s (**a**), O1s (**b**), and Ti2p (**c**) for Ti-6Al-4V samples in all studied conditions.

The semi-quantitative results from the high-resolution (HR) spectra of the C1s, O1s, and Ti2p peaks are illustrated in Figure 9. The fitted spectra of all elements of interest are found in the Supplementary Materials. The C1s spectrum (Figure 9a) exhibited a convolution of three peaks related to major C-C, C-O, and minor O=C-O bonding. The PEO treatment tended to increase the C-C bonding and decay that of C-O. The O1s spectrum (Figure 9b) also possessed three main components related to major O-H and some amounts of O-C and O-Metal bonding. Overall, the PEO treatment augmented the O-Metal component to the detriment of the O-C. Lastly, the Ti2p spectrum (Figure 9c) depicted mainly the Ti^4+^ chemical state (TiO_2_), the presence of Ti^3+^, which is related to sub-oxides (e.g., Ti_2_O_3_), and Ti^0^ (metallic bonding). Overall, the PEO-treated samples were mainly formed by the TiO_2_ bonded with organic and hydroxyl radical groups in the outer layer. According to Parfenov et al. [27], the PEO treatment is a useful tool to biofunctionalize the surface of Ti and its alloys, improving their chemical activity with peptides and organic molecules that trigger the cell mechanisms.

**Figure 10:**
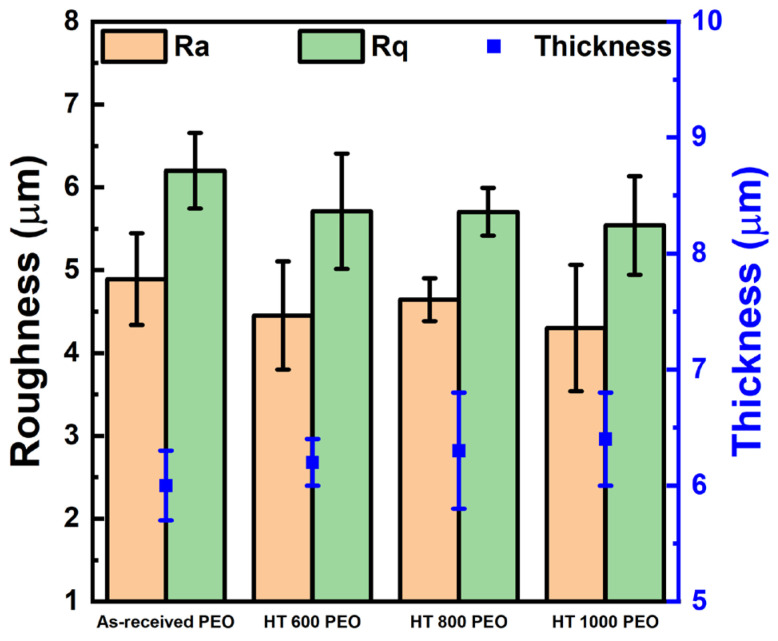
Roughness and thickness for PEO-treated Ti-6Al-4V samples.

Figure 10 presents the PEO-treated samples’ roughness (Ra and Rq) and thickness values. The roughness remained in the order of some micrometers, between 4 and 5 μm for Ra and 5 and 7 μm for Rq, while the thickness exhibited typical values of around 6–7 μm. A sensible difference in the values between the samples was undetected, indicating that the α and β phases did not play a role in these parameters. As is well known, micrometric surface roughness favors cell adhesion, growth, and proliferation, an important biological parameter to consider during implantation [28].

**Figure 11:**
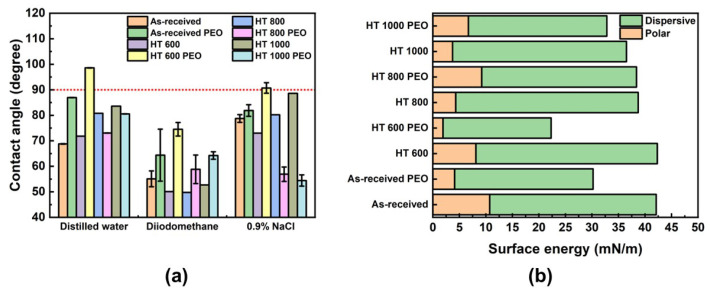
Wettability test: contact angle (**a**) and surface energy (**b**) for Ti-6Al-4V samples in all studied conditions. The dotted red line refers to the limit between hydrophilic and hydrophobic surfaces.

Figure 11a compares the contact angle values collected in distilled water, diiodomethane, and 0.9% NaCl with the corresponding surface energy values. Overall, the samples depicted hydrophilic behavior (contact angle lower than 90°) in all droplets except for the HT 600 PEO sample. The contact angle possessed the smallest values in diiodomethane without a clear tendency to vary with the α and β phases or the PEO treatment. Regarding the surface energy (Figure 11b), all samples depicted most of the dispersive component instead of the polar one, with the PEO treatment tending to diminish the total surface energy compared to the untreated material. According to Ponsonnet et al. [29], the dispersive component is related to the force interactions produced by non-polar radical groups, guaranteeing better cell proliferation along the surface.

**Figure 12:**
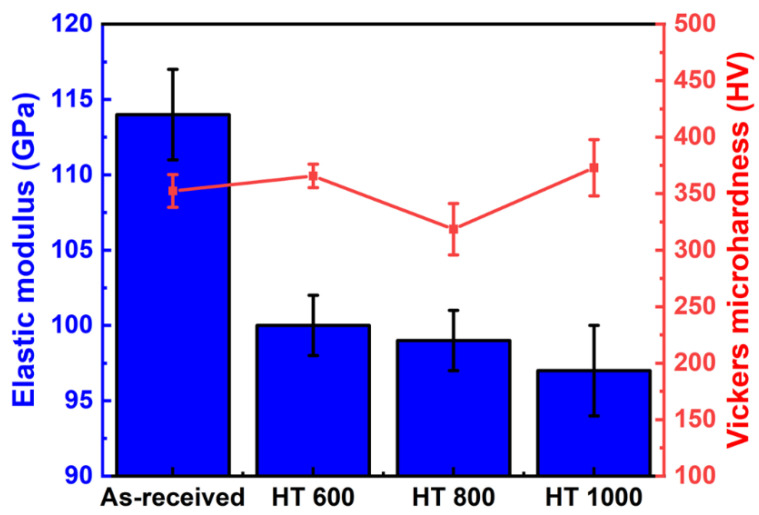
Selected mechanical properties for untreated Ti-6Al-4V samples.

Figure 12 compares the Vickers microhardness and elastic modulus only for the untreated samples since it was not possible to acquire the values for the PEO-treated samples precisely. The elastic modulus depicted a gradual decay with the heat treatments, resulting directly from the retained β phase formation to the detriment of the α phase. Regarding the Vickers microhardness, the values were higher than those of the commercially pure Ti grade 2 (~185 HV). However, there was no clear tendency for variation with the heat treatments, indicating that the solid-solution-strengthening and work-hardening mechanisms could be the main influence instead of the phase precipitation hardening. Overall, the combination of low elastic modulus and high hardness is useful for implants as it can attenuate the stress shielding effect and provide support for biomechanical loads [30]. Further evaluation of the mechanical properties by tensile tests can provide an in-depth view of the influence of the α/β phases and PEO treatment.

**Figure 13:**
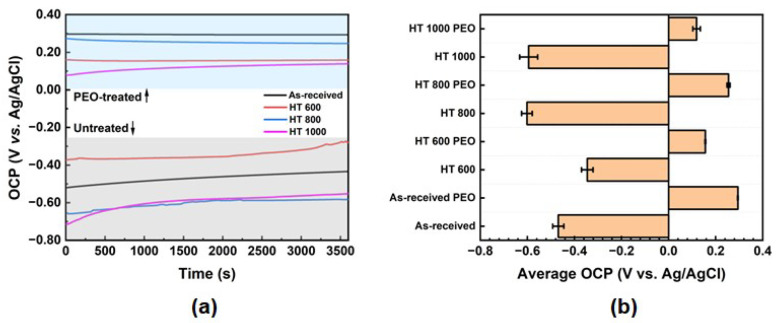
OCP results: OCP vs. time plot (**a**) and average OCP value (**b**) for Ti-6Al-4V samples in all studied conditions.

Figure 13 presents the OCP curves and a comparison of the average values. All samples presented a stable OCP curve during the evaluated time (Figure 13a), with a small tendency to increase, indicating the presence of a stable oxide layer. Furthermore, the PEO-treated samples showed more positive values than the untreated ones, confirming the favorable protective effect of the oxide layer grown on the surface. The average values (Figure 13b) remained positive for the PEO-treated samples, showing a remarkable anticorrosion ability of the PEO treatment compared to the heat treatments. The obtained values were similar to those of Garcia-Cabezón et al. [26], who investigated the anticorrosion properties of the Ti-6Al-4V alloy prepared by powder metallurgy and subjected to the same PEO treatment.

**Figure 14:**
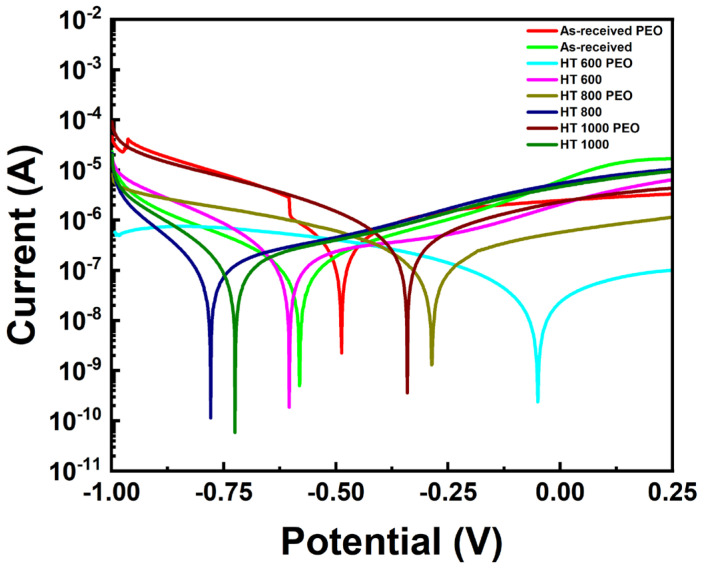
PDP results for Ti-6Al-4V samples in all studied conditions.

Figure 14 and Table 2 present the PDP curves and the calculated quantitative results. Overall, the PEO-treated samples, besides a similar corrosion current, exhibited a more positive corrosion potential than the untreated ones. The corresponding Rp value remained in the order of dozens of kΩ, with the HT 600 PEO sample possessing the highest value. In the same way, the PEO treatment acted more directly against the corrosion mechanisms than the heat treatments. According to Sobolev, Zinigrad, and Borodianskiy [31], the superior anticorrosion properties of the PEO-treated surfaces are due to a double electric layer formed in the coating, with the outer layer being porous and enriched with bioactive species and the inner layer dense and responsible for protecting the substrate.

**Figure 15:**
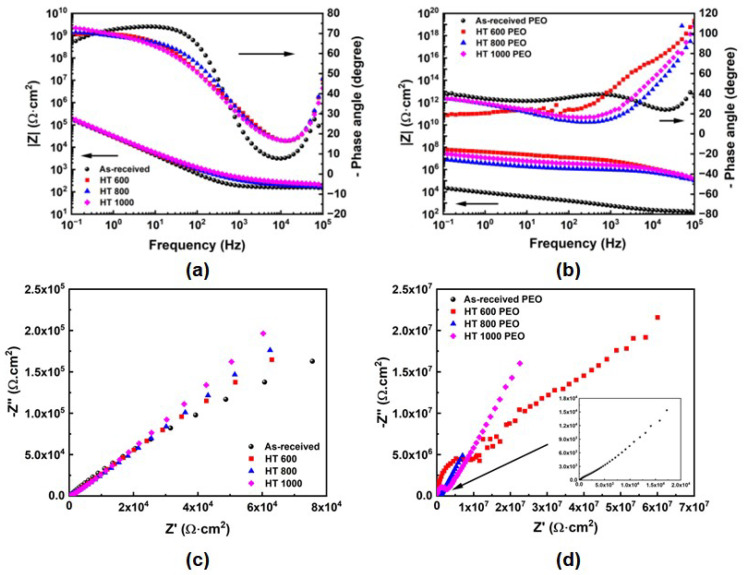
EIS results: Bode plots for the untreated (**a**) and PEO-treatment (**b**) Ti-6Al-4V samples and the corresponding Nyquist plots in (**c**,**d**).

The EIS curves, Bode and Nyquist plots are indicated in Figure 15. The phase angles for the untreated and PEO-treated samples (Figure 15a,b) were distinct, indicating different oxide layers on the surface. Furthermore, the impedance modulus (|Z|) at low frequencies (10^−1^ Hz) remained in the order of 10^9^ Ω·cm^2^ for the untreated samples and 10^10^–10^12^ Ω·cm^2^ for the PEO-treated ones, suggesting a better protective effect of the PEO coatings compared to the naturally formed passive layer. The Nyquist plot (Figure 15c) for the untreated sample depicted a clear semi-circle corresponding to one capacitive phase element, gradually increasing the radius with the augmenting of the β phase. In contrast, the PEO-treated samples (Figure 15d) depicted two semi-circles, suggesting two distinct capacitive phase elements with a larger radius than the previous one. Previous reports [32,33,34] indicated the presence of multilayers in the PEO coatings, which results in distinct elements in the electrical equivalent circuit in EIS spectra, giving beneficial responses to the anticorrosion properties.

**Figure 16:**
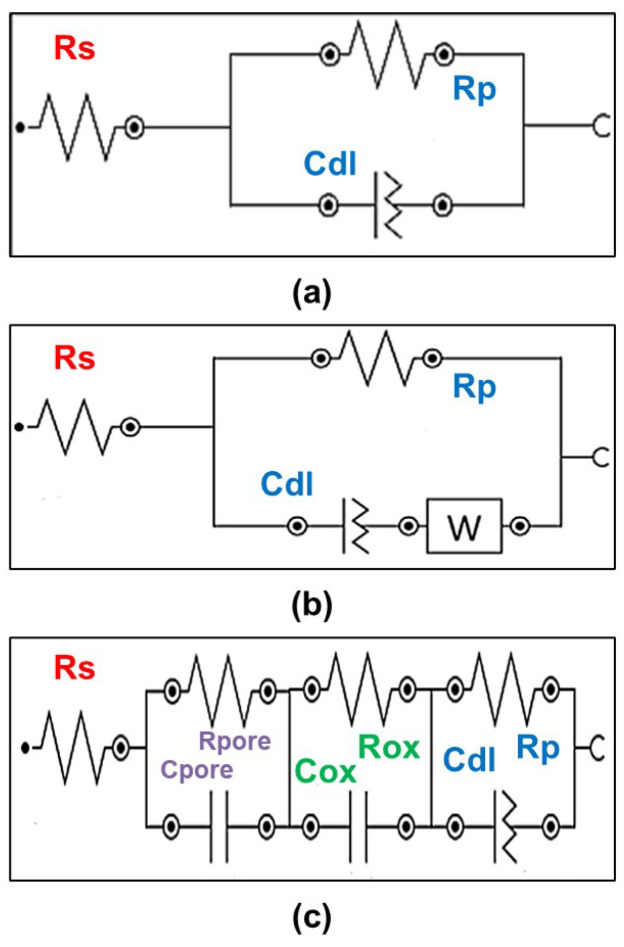
EEC diagrams of the EIS curves: (**a**) as-received sample; (**b**) HT 600, HT 800, HT 1000, and as-received PEO samples; (**c**) HT 600 PEO, HT 800, and HT 1000 PEO samples.

Figure 16 summarizes the EECs diagrams used to fit the EIS curves, and Table 3 and Table 4 show the corresponding quantitative values. A goodness-of-fitness value (χ^2^) below 1.0 was considered for a successful fitting. Overall, the untreated samples depicted a simple connection between the solution resistance (Rs) and a constant phase element (CPE) related to the metallic surface. The CPE component is characterized by a parallel configuration of the polarization resistance (Rp) and an imperfect capacitor (C_dl_) related to the protective capability and charge accumulation on the surface, respectively. The heat-treated samples also depicted a Warburg element (W), which is associated with diffusion processes into the surface. Thus, for the untreated samples, it is possible to conclude that the corrosion resistance came from a single passive layer naturally formed on the surface, with the α/β phases having minor effects on the surface chemical activity. On the other hand, the PEO-treated samples possessed a more complex EEC, composed of three CPE components. The CPEs components were related to the outer porous layer, inner dense layer, and the metallic surface, respectively. The corresponding resistive and capacitive elements for the porous (R_pore_ and C_pore_), dense (R_ox_ and C_ox_) oxide layers, and substrate (R_p_ and C_dl_) guarantee superior protection against the corrosion of the PEO-treated samples when compared to the untreated samples. It is worth pointing out that the capacitive element for the porous and dense oxide layer was a pure capacitor, which is related to a uniform and defect-free layer. Furthermore, the n/p value for the PEO-treated samples depicted is not so close to 1.0 as the untreated ones, indicating more resistive behavior, which is positive in avoiding surface degradation. According to the literature [35,36], the combination of the oxidative reactions and plasma discharges of the PEO treatment are the origins of the multi-layered EEC, being beneficial for protection against the corrosive environment.

## 4. Conclusions

This study evaluated the effect of distinct α/β phase proportions of the Ti-6Al-4V alloy on the growth mechanisms of the PEO treatment in a Ca- and P-rich electrolyte. From the results, it is possible to summarize the findings below:

- The α/β phase proportion changed with the temperature of the heat treatments, leading to α phase plates and β phase precipitate formation in the boundaries;
- All PEO-treated samples possessed a microsized porous surface of TiO_2_ with equal amounts of anatase and rutile;
- The amounts of Al and V were depleted in the oxide layer, while Ca and P were enriched. The chemical bonding detected in the outer layer gave evidence for the incorporation of calcium, phosphorus, absorbed water, and organic molecules;
- The PEO-treated samples possessed a microsized roughness and thickness, with hydrophilic contact angles and surface energy composed mostly of the dispersive component;
- The elastic modulus showed a gradual decay with the β phase precipitation, while the Vickers microhardness was exclusively affected by the alloying elements and the work-hardening effect;
- The OCP curves indicated a more stable protective layer for the PEO-treated samples than the bulk, while the PDP curves highlighted their superior corrosion resistance. The EIS indicated the presence of multiple protective elements in the oxide layer of the PEO-treated samples, which beneficially contributed to corrosion resistance. Further biological testing can provide a better view of the protective ability of the PEO treatment;
- The findings show that the α/β phase proportion considerably affected the mechanical properties of the Ti-6Al-4V alloy, while the PEO treatment had an effect on the anticorrosion properties and surface aspects. Thus, combining heat and PEO treatments makes it possible to handle the surface and bulk properties of this commercial biomaterial to provide superior performance for usage as biomedical implants.

## Figures and Tables

**Table 1:** Sample labeling.

Sample	Condition
As-received	Raw metal
HT 600	Heat-treated at 600 °C
HT 800	Heat-treated at 800 °C
HT 1000	Heat-treated at 1000 °C
As-received PEO	PEO-treated raw metal
HT 600 PEO	Heat-treated at 600 °C and PEO-treated
HT 800 PEO	Heat-treated at 800 °C and PEO-treated
HT 1000 PEO	Heat-treated at 1000 °C and PEO-treated

**Table 2:** Quantitative results from the PDP curves.

Sample	E_corr_ (mV)	j_corr_ (nA·cm^−2^)	R_p_ (kΩ)
As-received	−580	120	544
As-received PEO	−490	120	98
HT 600	−600	180	252
HT 600 PEO	−50	70	2380
HT 800	−780	120	381
HT 800 PEO	−290	260	565
HT 1000	−720	140	402
HT 1000 PEO	−340	640	164

**Table 3:** Quantitative results from fitting the EIS curves for the untreated samples.

Parameters	As-Received	HT 600	HT 800	HT 1000
Rs (MΩ·cm^2^)	1.52 × 10^−4^	1.55 × 10^−4^	1.68 × 10^−4^	2.18 × 10^−4^
Rp (MΩ·cm^2^)	7.63 × 10^−3^	1.29	4.69	7.63
C_dl_ (µMho·s^n^)	7.31	8.65	8.26	7.88
n/p	0.842	0.87	0.821	0.856
W (µMho·s^1/2^)	-	69.7	99.0	57.8
χ^2^	0.053511	0.025978	0.03358	0.021966

**Table 4:** Quantitative results from fitting the EIS curves for the PEO-treated samples.

Parameters	As-Received PEO	HT 600 PEO	HT 800 PEO	HT 1000 PEO
Rs (MΩ·cm^2^)	1.2 × 10^−4^	3.58 × 10^−2^	3.75 × 10^−2^	9.36 × 10^−3^
Rpore (MΩ·cm^2^)	-	6.59	8.44 × 10^−1^	2.06
Cpore (pF)	-	18.5	22.6	13.9
Rox (MΩ·cm^2^)	-	31.9	8.86	27.3
Cox (nF)	-	32.4	233	58.7
Rp (MΩ·cm^2^)	5.03 ×10^−3^	36.6	4.07	11.2
C_dl_ (µMho·s^n^)	14.7	3.49 × 10^−3^	75.2 × 10^−3^	17.7 × 10^−3^
n/p	0.557	0.600	0.559	0.559
W (µMho·s^1/2^)	46.6	-	-	-
χ^2^	0.027026	0.41719	0.29647	0.46814

## Data Availability

The original contributions presented in this study are included in the article and Supplementary Materials. Further inquiries can be directed to the corresponding author.

## Supplementary Materials

The following supporting information can be downloaded at: https://www.mdpi.com/article/10.3390/ma18050955/s1, Figure S1: High-resolution XPS spectra for aluminum; Figure S2: High-resolution XPS spectra for vanadium; Figure S3: High-resolution XPS spectra for calcium; Figure S4: High-resolution XPS spectra for phosphorus; Figure S5: High-resolution XPS spectra for carbon; Figure S6: High-resolution XPS spectra for oxygen. Auger peak (Na KLL) is an adventitious impurity; Figure S7: High-resolution XPS spectra for titanium; Table S1: Fitting details for aluminum; Table S2: Fitting details for calcium; Table S3: Fitting details for phosphorus; Table S4: Fitting details for carbon; Table S5: Fitting details for oxygen; Table S6: Fitting details for titanium.

## Abbreviations

The following abbreviations are used in this manuscript:

PEO	Plasma electrolytic oxidation
MAO	Micro-arc oxidation
XRD	X-ray diffraction
OM	Optical microscopy
SEM	Scanning electron microscopy
FTIR	Fourier-transform infrared spectroscopy
XPS	X-ray photoelectron spectroscopy
OCP	Open-circuit potential
PDP	Potentiodynamic polarization
EIS	Electrochemical impedance spectroscopy
EEC	Equivalent electrical circuit
|Z|	Impedance modulus
Z′	Real component of the impedance
Z″	Imaginary component of the impedance
CPE	Constant phase element
Rs	Solution resistance
Rp	Polarization resistance
Cdl	Imperfect capacitor
Rpore	Polarization resistance of the porous layer
Cpore	Pure capacitor of the porous layer
Rox	Polarization resistance of the inner oxide layer
Cox	Pure capacitor of the inner oxide layer
W	Warburg element
n/p	Exponent of the imperfect capacitor
χ2	Goodness-of-fitness factor
