# Supplementary material for: Effect of Bulk Phase Composition on the Growth of PEO Coatings on the Biomedical Ti-6Al-4V Alloy
Source: Materials (Basel). 2025 Feb 21;18(5):955. doi: 10.3390/ma18050955 (PMC11901143; doi:10.3390/ma18050955)
Supplement: Supplementary file 1 [file materials-18-00955-s001.zip › materials-3460963-supplementary.pdf]

Supplementary material

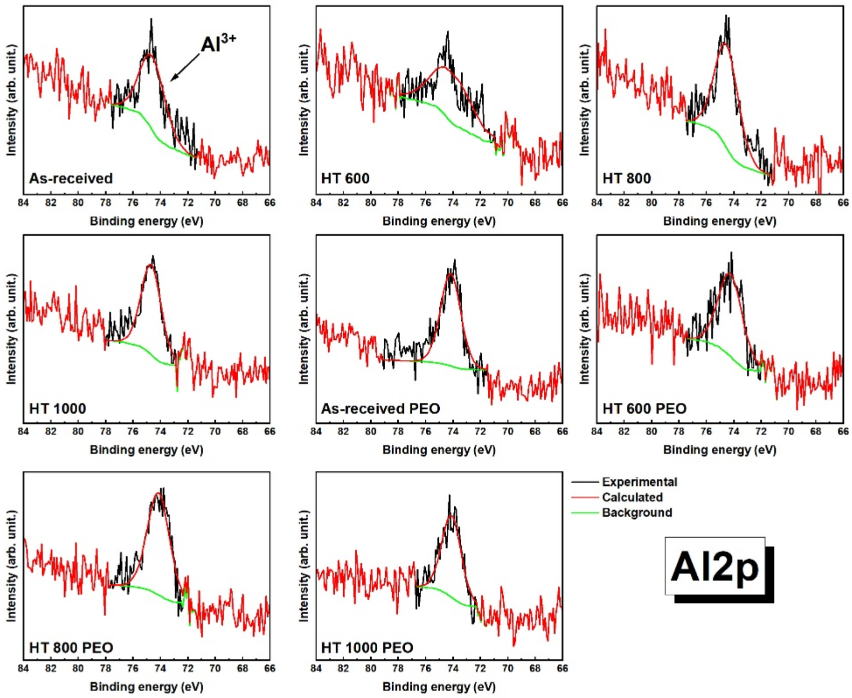

Figure S1: High-resolution XPS spectra for aluminum.

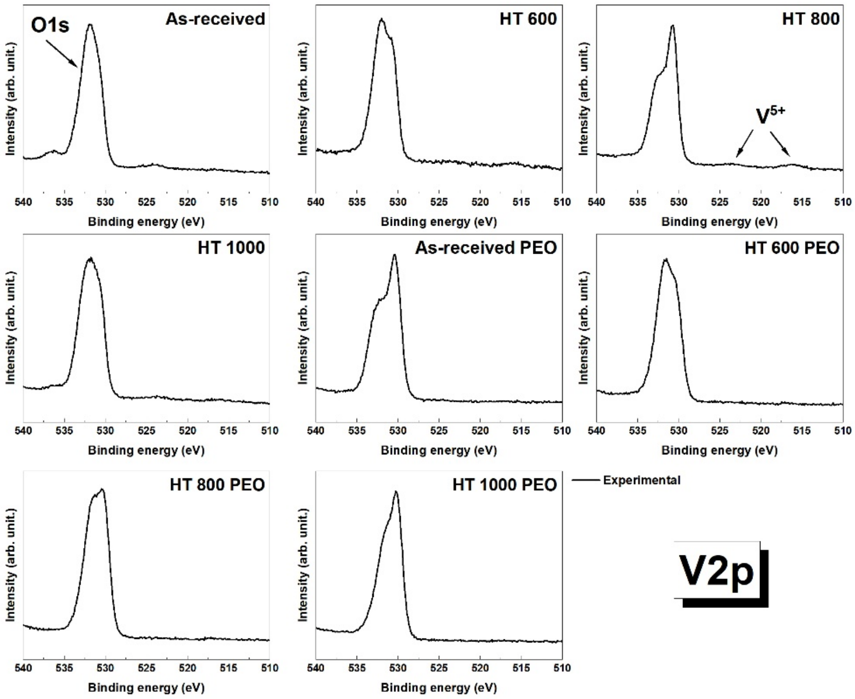

Figure S2: High-resolution XPS spectra for vanadium.

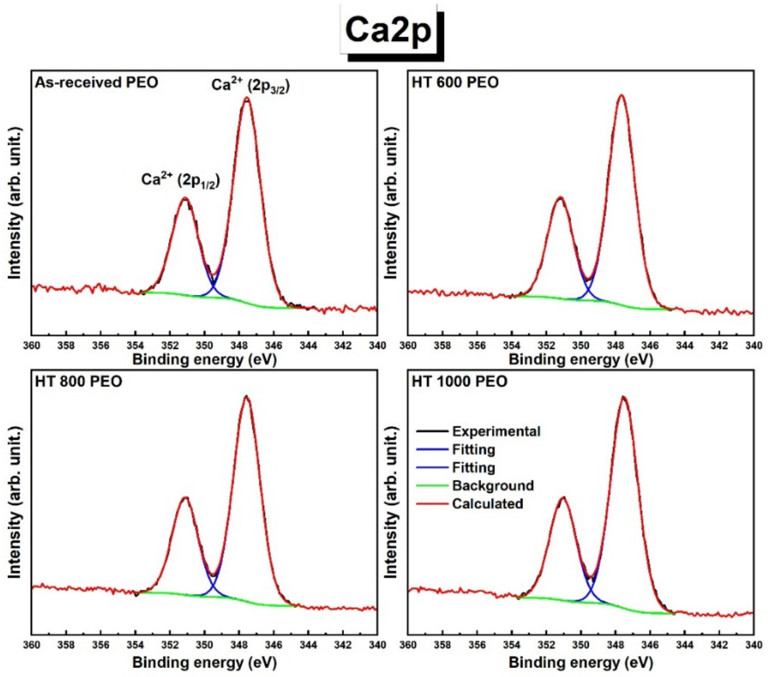

Figure S3: High-resolution XPS spectra for calcium.

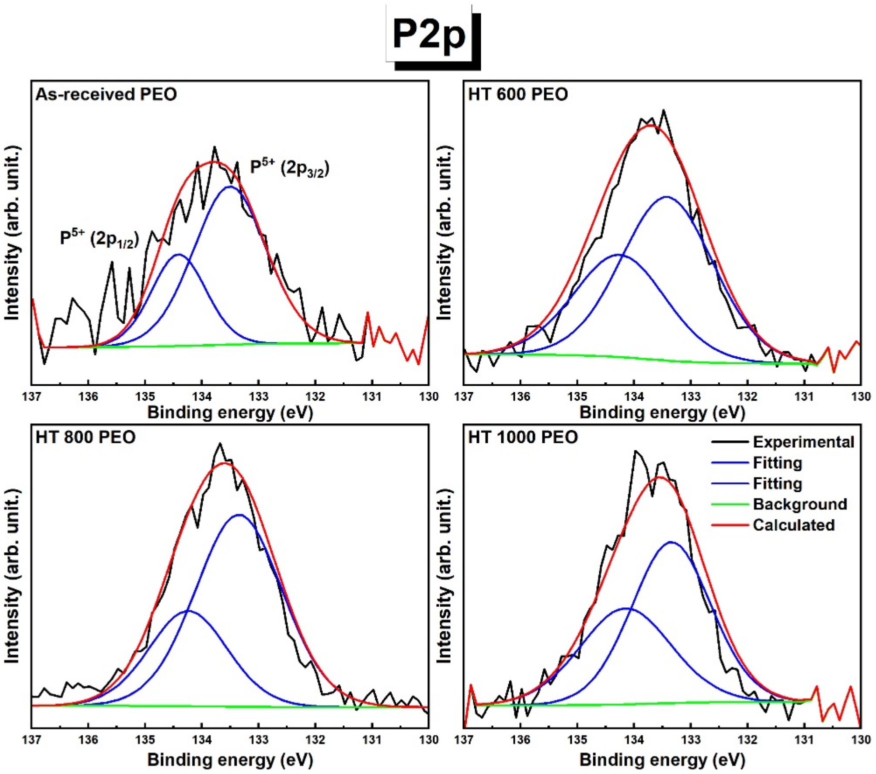

Figure S4: High-resolution XPS spectra for phosphorus.

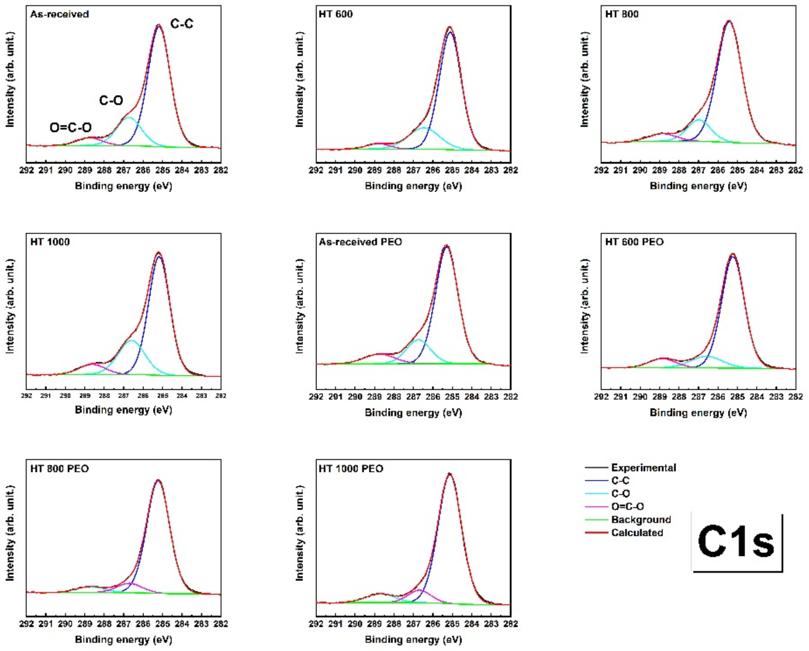

Figure S5: High-resolution XPS spectra for carbon.

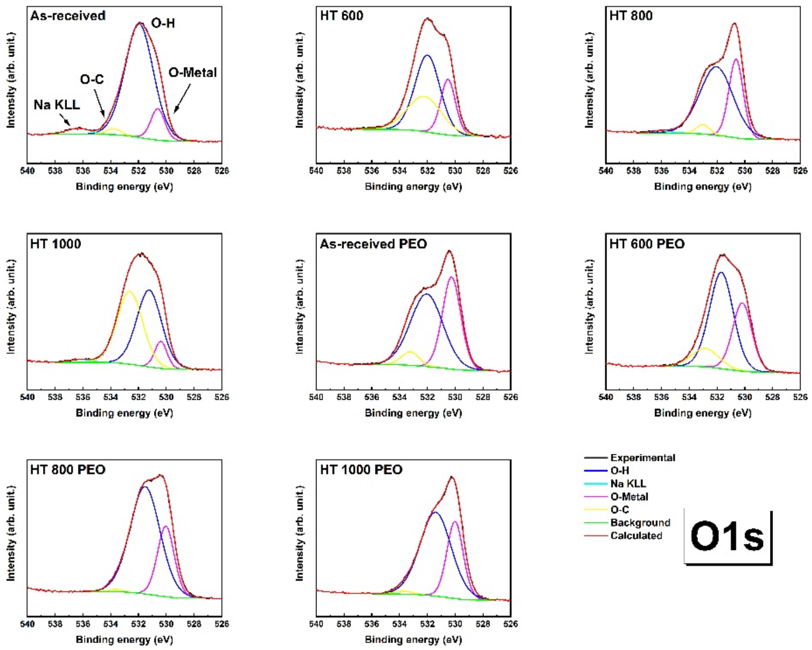

Figure S6: High-resolution XPS spectra for oxygen. Auger peak (Na KLL) is an adventitious impurity.

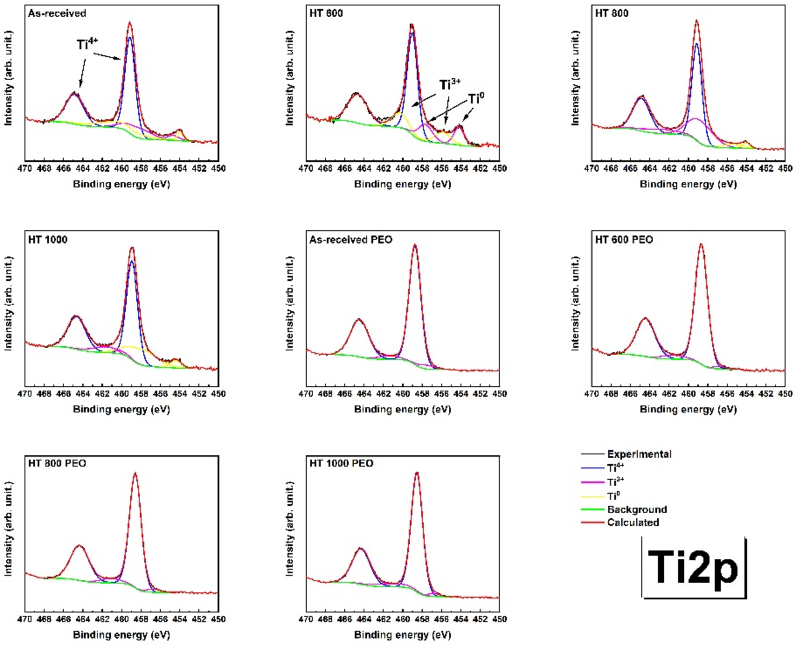

Figure S7: High-resolution XPS spectra for titanium.

Table S1: Fitting details for aluminum.

Sample	Al^{3+}	
	Peak position (eV)	Residual
As-received	74.58	1.16767

**HT 600**	74.14	1.0175
**HT 800**	74.51	0.9991
**HT 1000**	74.67	0.9302
**As-received PEO**	74.17	1.1762
**HT 600 PEO**	74.24	1.0267
**HT 800 PEO**	74.15	1.0336
**HT 1000 PEO**	74.10	1.0320

Table S2: Fitting details for calcium.

Sample	Ca^{2+}		Residual
	$2p_{3/2}$ Peak position (eV)	$2p_{1/2}$ Peak position (eV)	
**As-received PEO**	347.55	351.12	1.1184
**HT 600 PEO**	347.65	351.20	1.0138
**HT 800 PEO**	347.56	351.13	1.0749
**HT 1000 PEO**	347.49	351.05	1.2585

Table S3: Fitting details for phosphorus.

Sample	P^{5+}		Residual
	$2p_{3/2}$ Peak position (eV)	$2p_{1/2}$ Peak position (eV)	
**As-received PEO**	133.51	134.41	1.2431
**HT 600 PEO**	133.41	134.25	1.4896
**HT 800 PEO**	133.35	134.24	1.4242
**HT 1000 PEO**	133.35	134.15	1.6127

Table S4: Fitting details for carbon.

Sample	C-C	C-O	O=C-O	Residual
	Peak position (eV)	Peak position (eV)	Peak position (eV)	
**As-received**	285.18	286.73	288.75	1.3610
**HT 600**	285.11	286.46	288.82	1.7267
**HT 800**	285.42	286.98	288.84	1.0241
**HT 1000**	285.18	286.60	288.62	1.7367
**As-received PEO**	285.29	286.75	288.74	1.1405
**HT 600 PEO**	285.23	286.60	288.87	1.6933
**HT 800 PEO**	285.23	286.72	288.72	1.6602
**HT 1000 PEO**	285.13	286.69	288.70	1.8858

Table S5: Fitting details for oxygen.

Sample	O-C (eV)	O-H (eV)	O-Metal (eV)	Residual
	Peak position (eV)	Peak position (eV)	Peak position (eV)	
**As-received**	533.79	531.94	530.61	1.5950
**HT 600**	532.21	531.99	530.51	0.7520

**HT 800**	533.04	532.04	530.63	1.2024
**HT 1000**	532.62	531.23	530.39	0.8755
**As-received PEO**	533.23	532.04	530.25	0.9911
**HT 600 PEO**	532.85	531.69	530.18	0.9726
**HT 800 PEO**	533.62	531.52	530.03	0.9265
**HT 1000 PEO**	533.76	531.40	530.01	1.0190

Table S6: Fitting details for titanium.

Sample	Ti^{4+}		Ti^{3+}		Ti^{0}		Residual
	Peak position (eV)	Peak position (eV)	Peak position (eV)	Peak position (eV)	Peak position (eV)	Peak position (eV)	
**As-received**	459.13	464.85	454.70	461.40	453.98	460.25	0.8999
**HT 600**	459.01	464.63	454.19	461.04	454.70	460.31	1.2929
**HT 800**	459.13	464.85	458.77	462.85	454.10	460.43	0.8822
**HT 1000**	458.93	464.64	457.94	461.65	454.39	460.49	0.9022
**As-received PEO**	458.74	464.46	457.54	461.39	-	-	0.9238
**HT 600 PEO**	458.67	464.42	456.82	461.56	-	-	0.8915
**HT 800 PEO**	458.59	464.33	456.75	461.07	-	-	0.8715
**HT 1000 PEO**	458.54	464.30	456.83	461.00	-	-	0.9335
